# Vimentin-Mediated Steroidogenesis Induced by Phthalate Esters: Involvement of DNA Demethylation and Nuclear Factor κB

**DOI:** 10.1371/journal.pone.0146138

**Published:** 2016-01-08

**Authors:** Yuan Li, Yanhui Hu, Congcong Dong, Hongchao Lu, Chang Zhang, Qi Hu, Shifeng Li, Heng Qin, Zhong Li, Yubang Wang

**Affiliations:** The Key Laboratory of Modern Toxicology, Ministry of Education, School of Public Health, Nanjing Medical University, Nanjing, 211166, China; UMR INSERM U866, FRANCE

## Abstract

Di-n-butyl phthalate (DBP) and its active metabolite, monobutyl phthalate (MBP) are the most common endocrine disrupting chemicals. Many studies indicate that high-doses of DBP and/or MBP exhibit toxicity on testicular function, however, little attention have been paid to the effects of low levels of DBP/MBP on steroidogenesis. As we all know, the steroidogenic acute regulatory protein (StAR) is a key regulator involved in the steroidogenesis. Here we found that, in addition to StAR, MBP/DBP increased the steroidogenesis by a cytoskeletal protein, vimentin. Briefly, in murine adrenocortical tumor (Y1) and the mouse Leydig tumor (MLTC-1) cells, vimentin regulated the secretion of progesterone. When these two cells were exposure to MBP, the DNA demethylation in the vimentin promoter was observed. In addition, MBP also induced the activation of nuclear factor kappa B (NF-κB, a transcriptional regulator of vimentin). These two processes improved the transcriptional elevation of vimentin. Knockdown of NF-κB/vimentin signaling blocked the DBP/MBP-induced steroidogenesis. These *in vitro* results were also confirmed via an *in vivo* model. By identifying a mechanism whereby DBP/MBP regulates vimentin, our results expand the understanding of the endocrine disrupting potential of phthalate esters.

## Introduction

Endocrine disrupting chemicals (EDCs) are widespread environmental substances that have been introduced by man and may influence the endocrine system in a harmful manner [1]. Phthalate esters are a large group of industrial chemicals used mainly as plasticizers and solvents, and the annual global use of phthalates is estimated to exceed 3 million metric tons [2]. As there is no covalent bond between the phthalates and plastics in which they are mixed, they can leach out, migrate or gas out from the plastic to the external environment [3, 4]. So, people may be exposed to phthalates through a variety of sources, such as foodstuff, water, air, dust and the use of consumer and personal-care products [5].

Di-n-butyl phthalate (DBP), one of the most dominant phthalate esters, is widely used as a plasticizer in polyvinyl chloride products, cosmetics, and other personal care products [6]. DBP and its major metabolite, monobutyl phthalate (MBP), are commonly detected in a variety of biological samples [7]. Experimental evidences suggest that high-levels of DBP induce the toxicological effects on testicular function, which causes the reproductive injury, and decreases the circulating hormone concentrations [8, 9]. However, the effects of low-levels of DBP and/or MBP on the testicular function and steroidogenesis remain unclear.

In our previous study, we found a biphasic dose–response effect induced by DBP on pubertal rat. High-levels of DBP attenuated the circulating testosterone concentrations, while low-levels of DBP elevated the circulating testosterone concentrations; Further, by using two-dimension electrophoresis, we identified that vimentin was the significantly altered protein under the DBP exposure [10]. Studies indicate that vimentin is a key bridge between cholesterol and mitochondria [11, 12]. Based on these findings, we hypothesize that low-levels of DBP/MBP increase the steroidogenesis by vimentin. So, in our present study, we construct the *in vitro* and *in vivo* DBP/MBP-exposure models, and elucidate whether vimentin is a key target protein in the regulation of steroidogenesis.

## Materials and Methods

### Ethics Statement

This study was performed according to a protocol approved by the Nanjing Medical University Institutional Animal Care and Use Committee, and animals were treated humanely and with regard for alleviation of suffering.

### Chemicals

DBP and MBP were purchased from Tokyo Kasei Kogyo Co Ltd. (Tokyo, Japan). Human chorionic gonadotrophin (hCG) and forskolin were obtained from Sigma (St. Louis, MO, USA). RPMI 1640 medium, fetal bovine serum (FBS), streptomycin sulfate, antibiotic penicillin G sodium (10,000 U/ml), and phosphate-buffered saline with Ca^2+^ and Mg^2+^ were obtained from Gibco (Grand Island, NY, USA). S-adenosylmethionine (SAM) was purchased from New England BioLabs (Ipswich, MA, USA). All other chemicals used were of analytical grade.

### Cell culture

Murine Y1 adrenocortical tumor cells (Y1) and the mouse Leydig tumor cells (MLTC-1) were obtained from Cell Institute of Shanghai, Chinese Academy of Sciences (Shanghai, China). These cells were cultured in RPMI-1640 medium containing 100 IU/ml penicillin, 100 IU/ml streptomycin, and 10% FBS at 5% CO_2_ in 37°C. A mycoplasma stain assay Kit (Beyotime, Haimeng, China) was used for mycoplasma testing to rule out the possibility of cryptic contamination.

### Animals and treatment

Male Sprague–Dawley rats approximately 4 weeks old were purchased from Zhejiang Laboratory Animal Center (certification No. 0006505) and housed under controlled temperature (22 ± 2°C), lighting (12-h light and 12-h dark cycle) and relative humidity (40%–70%). A soy-free breeding diet and reverse-osmosis water were provided ad libitum. For siRNA injection, the rats were anesthetized with sodium pentobarbital, then the testes were exteriorized through abdominal incision. Vimentin-siRNA 5’-GAGUCAAACGAGUACCGGAtt-3’, RelA-siRNA 5’-AAUGUCUUCUUUCUGCACCdTdT-3’, and Con-siRNA 5’-UACGUACUAUCGCGCGGAUdTdT-3’ were synthesized by Ribobio. Co (Guangzhou, China). Approximately 10 nM of siRNA was injected into the interstitial tissue of testis. The mice were orally administered with DBP at the doses of 0 or 1 mg/kg·day for 30 days. At the end of then, the serum testosterone level was determined as described below, and the animals were sacrificed, and the tissue DNAs, RNAs, and proteins were collected for further experiments.

### Steroidogenesis assay

For progesterone determination, cells were plated at a density of 5×10^4^ cells/ml in 24-well plates for 24 h. Then they were treated with MBP in the presence of hCG (MLTC-1) or forskolin (For) for another 24 h. The media were kept to quantitate progesterone (P4) concentration by radio-immuno analysis. For the determination of serum testosterone, the rat serum was prepared by centrifuging at 2000g at 4°C for 10 min followed by the measurement with Coat-A-Count radio-immunoassay kits (Beijing North Institute of Biological Technology, China). No lipoproteins or serum included in the incubations when steroid production was measured.

### Determination of cell viability

A total of 2×10^3^ cells were seeded in 96-well plates. At the time of next day, they were treated by different concentrations of MBP, respectively. Then, such cells were incubated with 20.0 μl of CCK-8 solution (Dojindo Molecular Technologies, Inc, Kumamoto, Japan) for another 4 h. The absorbance at 450 nm was measured with a multi-well plate reader (Model 680, Bio-Rad, USA).

### Western blots

Cell lysates were separated by 10% sodium dodecyl sulfate-polyacrylamide gel electrophoresis, followed by transferring to polyvinylidene fluoride membranes (Millipore, Billerica, USA). Antibodies used were StAR, vimentin, phosphorylated inhibitor of nuclear factor kappa-B kinase subunit beta (p-IKKβ-Ser-180), phosphorylated inhibitor of nuclear factor kappa-B subunit alpha (p-IκBα-Ser-32), RelA, and p-RelA-Ser-536 (Cell Signaling Technology, Beverly, MA, 1: 1000 dilution), P450scc, 3β-HSD, DNA methyltransferase 1 (DNMT1), DNMT3a, DNMT3b, and Flag (Santa Cruz, CA, USA, 1: 200 dilution), glyceraldehyde 3-phosphate dehydrogenase (GAPDH), β-actin and tubulin (Sigma, 1: 1000 dilution). The immune complexes were detected by enhanced chemiluminescence (Cell Signaling Technology). For densitometric analyses, the bands were measured by the Eagle Eye II imaging system.

### Quantitative real-time polymerase chain reaction (qRT-PCR)

Total cellular RNA was isolated using Trizol (Invitrogen, Carlsbad, USA) according to the manufacturer’s recommendations. Then, 2 μg RNA was transcribed into cDNA using AMV Reverse Transcriptase (Promega). Primers used were: vimentin (F), 5’-CTGCTTCAAGACTCGGTGGAC-3’ and vimentin (R), 5’-ATCTCCTCCTCGTACAGGTCG-3’. qRT-PCR was performed using the MaximaTM SYBR Green/ROX qPCR Master Mix (Fermentas, Waltham, MA, USA), on the Applied Biosystems 7300HT machine.

### DNA methylation analysis

Cellular or tissue DNA was isolated using DNA purification kits (Qiagen, Germantown, MD, USA). The genomic DNA was modified with sodium bisulfite using the EpiTect Kit (Qiagen). DNA methylation was analyzed using a SYBR Green-based quantitative methylation-specific PCR (qMSP) as described previously. Primers used were: methylated sense 5’- CGGCGGGATAGTAGGGCGCG-3’ and antisense 5’- GGTAAGTCGATGGATAGAGGCG -3’; unmethylated sense 5’- TGGTGGGATAGTAGGGTGTG-3’ and antisense 5’- GGTAAGTTGATGGATAGAGGTG -3’. Briefly, 1 μl of bisulfite-treated DNA template was mixed with 10 μl of 2 × Power SYBR Green PCR Master Mix (Applied Biosystems) and a pair of primers in a final concentration of 400 nM. The PCR conditions included initial incubation at 50°C for 2 min, denaturing at 95°C for 10 min, and 40 cycles of denaturing at 95°C for 15 s and annealing at 60°C for 1 min.

### Cell transfection

Vimentin-siRNA 5’-GGAGAGCAGGAUUUCUCUGtt-3’, RelA-siRNA, and Con-siRNA (Ribobio. Co) were used in transfection experiments at 10 nM, while the pcDNA 3.1-vimentin-Flag construct (GeneRay. Co, Shanghai, China) was used at the quality of 5 μg. MLTC-1cells and Y1 cells were transiently transfected using the Lipofectamine 2000 reagent (Invitrogen) according to the manufacturer’s instructions. After 12 h of transfection, the medium was replaced, and the cells were treated for further experiments.

### Luciferase reporter assay

The pGL3-vimentin-Luc construct was purchased from Ribobio. Co. The plasmid phRL-tk containing the Renilla luciferase gene was purchased from Promega. Briefly, MLTC-1 cells were plated in 24-wells cell culture dishes for 24 h. Con-siRNA or RelA-siRNA was co-transfected with the reporter constructs respectively, by using Lipofecamine 2000 reagent (Invitrogen) according to the manufacturer’s protocol. After an incubation period of 12 h, the cells were lysed with passive lysis buffer (Promega), and the lysates were analyzed immediately with a 96-well plate luminometer (Berthold Detection System, Pforzheim, Germany).

### Statistics

Data were presented as the means ± SD. A Student’s t test, and a one-way analysis of variance (ANOVA) followed by Dunnett’s t test were used to assess significant differences between groups. P values <0.05 were considered statistically significant.

## Results

### Effects of MBP on the progesterone secretion

**Fig 1:**
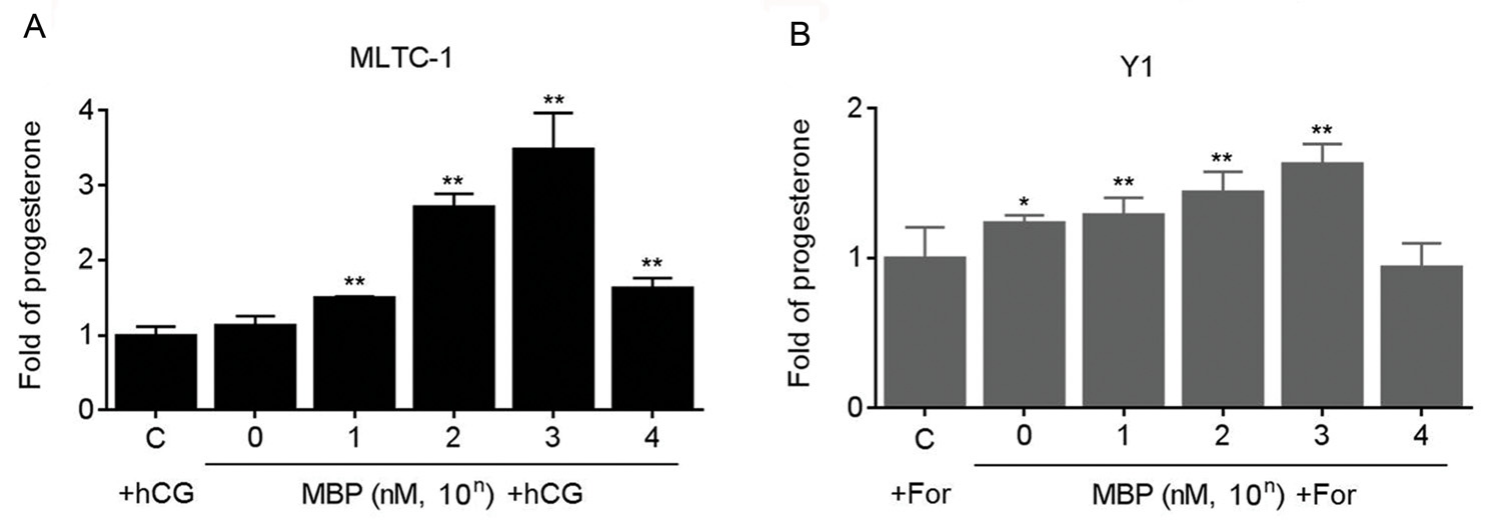
Effects of MBP on the progesterone secretion. (A) MLTC-1 and (B) Y1 cells were exposed to 0 ~ 10^4^ nM MBP in the presence of 100 U/L hCG or 10 μM forskolin for 24 h respectively, the secretion of progesterone was determined in triplicate. The amounts of progesterone measured in control cells were MLTC-1: 8.85±0.21 ng/ml, Y1:8.01±0.32 ng/ml, respectively. *p<0.05 and **p<0.01 compared with cells exposed to hCG or forskolin alone.

The mouse MLTC-1 and Y1 cells are the classical cell models, which are used for investigating the hormone secretion *in vitro* [13, 14]. We firstly determined the effects of MBP on the cell viabilities. There were decreases of viabilities only in the cells exposed to 10^6^ nM MBP (S1 Fig). Next, we investigated the effects of MBP on the progesterone secretion. It has been reported that there is very little progesterone secretion in these two cells under the routine culture condition. So two classical agonists, hCG and forskolin were used to stimulate the progesterone secretion [13, 14]. Here, at the doses range from 1 ~ 10^3^ nM, MBP elevated the progesterone secretion in a dose-dependent manner (Fig 1A and 1B). So the concentration of 1000 nM MBP was chosen for further investigation.

### Effects of MBP and hCG/For on the expressions of StAR and vimentin

**Fig 2:**
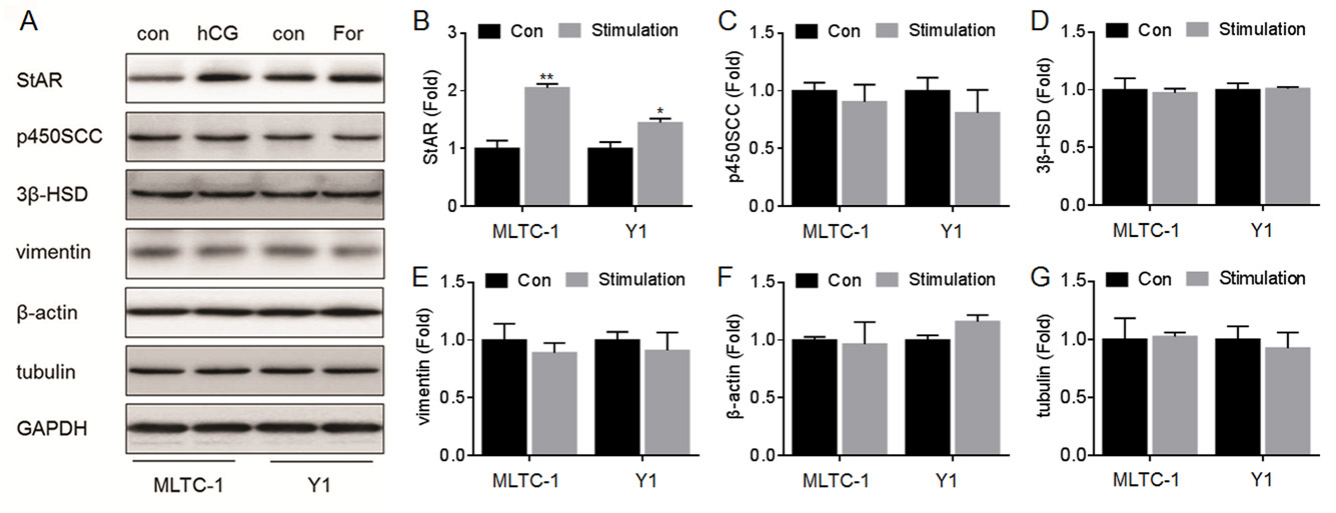
Effects of hCG/For on the expressions of StAR, p450scc, 3β-HSD, vimentin, β-actin, and tubulin. MLTC-1 and Y1 cells were exposed to 100 U/L hCG or to 10 μM forskolin for 24 h, respectively. (A) Western blots analysis and relative protein levels of (B) StAR, (C) p450SCC, (D) 3β-HSD, (E) vimentin, (F) β-actin, and (G) tubulin. *p<0.05 and **p<0.01 compared with medium control cells.

**Fig 3:**
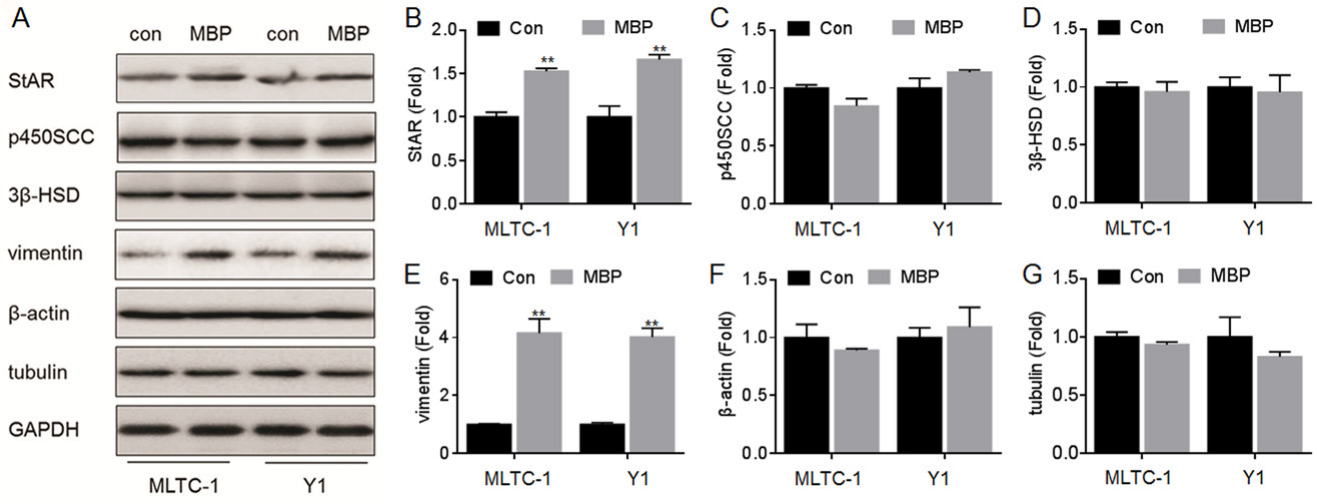
Effects of MBP on the expressions of StAR, p450scc, 3β-HSD, vimentin, β-actin, and tubulin. MLTC-1 and Y1 cells were treated by 1000 nM MBP for 24 h. (A) Western blots analysis and relative protein levels of (B) StAR, (C) p450SCC, (D) 3β-HSD, (E) vimentin, (F) β-actin, and (G) tubulin. **p<0.01 compared with medium control cells.

**Fig 4:**
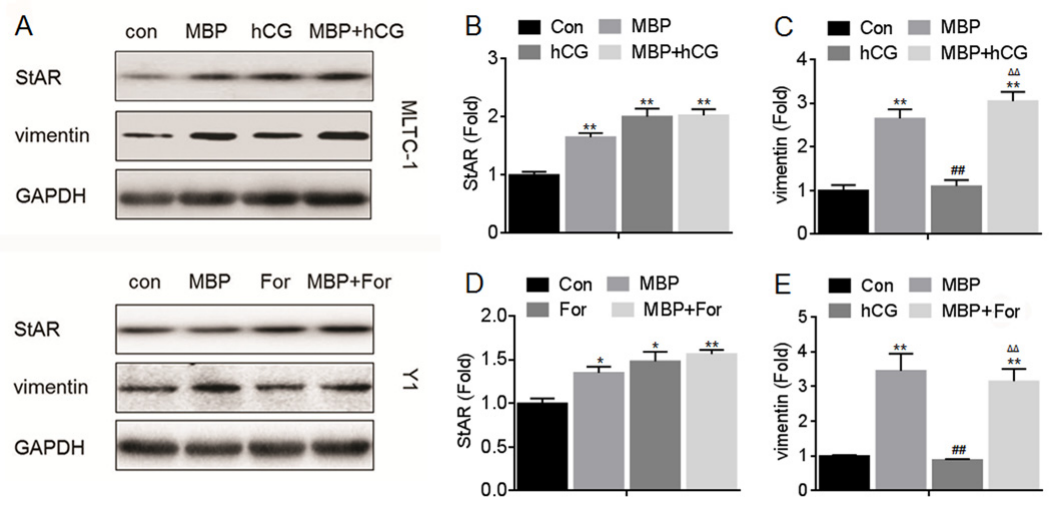
Effects of MBP and hCG/For on the expressions of StAR, p450scc, 3β-HSD, vimentin, β-actin, and tubulin. MLTC-1 and Y1 cells were exposed to1000 nM MBP in the absence or presence of 100 U/L hCG or 10 μM forskolin for 24 h, respectively. (A) Western blots analysis and relative protein levels of (B) StAR, (C) p450SCC, (D) 3β-HSD, (E) vimentin, (F) β-actin, and (G) tubulin. **p<0.01 compared with medium control cells; ^##^p<0.01 compared with cells treated by MBP alone; ^ΔΔ^p<0.01 compared with cells treated by hCG or For alone.

In the hormone synthesis and secretion signal, several classical proteins such as StAR, p450scc and 3β-HSD are involved in [15, 16]. Moreover, some cytoskeletal proteins such as β-actin and tubulin play an important role in the transportation of cholesterol, a hormone prosoma [17]. Further, we previously showed that vimentin, a cytoskeletal protein, might be involved in the hormone synthesis *in vivo* [10]. Here, we firstly investigated the effects of hCG, forskolin, or MBP on the expressions of these proteins. Our data showed that, the hCG and/or forskolin significantly improved the expression of StAR but not vimentin (Fig 2A, 2B and 2E); Interestingly, MBP significantly elevated the expressions of both vimentin and StAR (Fig 3A, 3B and 3E). However, there was no significantly effect of hCG, forskolin, and MBP on the expressions of p450scc, 3β-HSD, β-actin, and tubulin (Figs 2 and 3C, 3D, 3F and 3G). We then determined the effects of MBP on the expressions of StAR and vimentin in the presence of hCG and/or forskolin. There were increased expressions of StAR (Fig 4A, 4B and 4D) and vimentin (Fig 4A, 4C and 4E) in cells exposed to MBP alone or in combination with hCG and/or forskolin. So we hypothesized that in addition to StAR, vimentin, a cytoskeletal protein, was involved in the MBP-induced increased synthesis/secretion of steroid hormone.

### Effects of vimentin on the MBP-induced progesterone secretion

**Fig 5:**
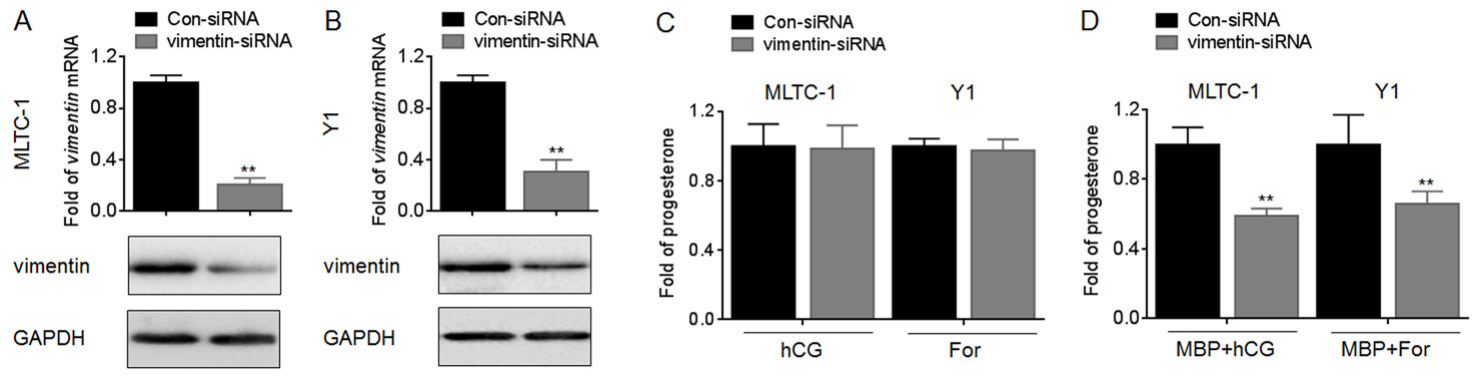
Effects of vimentin on the MBP-induced progesterone secretion. (A and B), MLTC-1 and Y1 cells were transfected by con-siRNA or vimentin-siRNA for 12 h, respectively, qRT-PCR (top) and Western blots (bottom) analysis of the expressions of vimentin mRNA and protein. (C and D), after MLTC-1 and Y1 cells were transfected by con-siRNA or vimentin-siRNA for 12 h, they were exposed to100 U/L hCG or 10 μM forskolin in the absence or presence of 1000 nM MBP for 24 h, respectively. The secretion of progesterone was determined in triplicate. The amounts of progesterone measured in control cells were MLTC-1 (hCG): 8.32±0.22 ng/ml, MLTC-1 (hCG+MBP): 10.10±0.42 ng/ml, Y1 (For): 8.64±0.33 ng/ml, and Y1 (For+MBP): 10.31±0.21 ng/ml respectively. **p<0.01 compared with cells transfected by con-siRNA.

To further confirm our hypothesis, we used siRNA to block the expression of vimentin (Fig 5A and 5B). Our data showed that, knockdown of vimentin did not affect the progesterone secretion in MLTC-1 and Y1 cells treated by hCG or For alone (Fig 5C). However, knockdown of vimentin significantly decreased the secretion of progesterone in MLTC-1 and Y1 cells treated by hCG or For in the presence of MBP (Fig 5D). These results indicated that knockdown of vimentin decrease the steroid production induced by MBP.

### MBP improved the expression of vimentin by inducing the DNA demethylation and NF-κB activation

**Fig 6:**
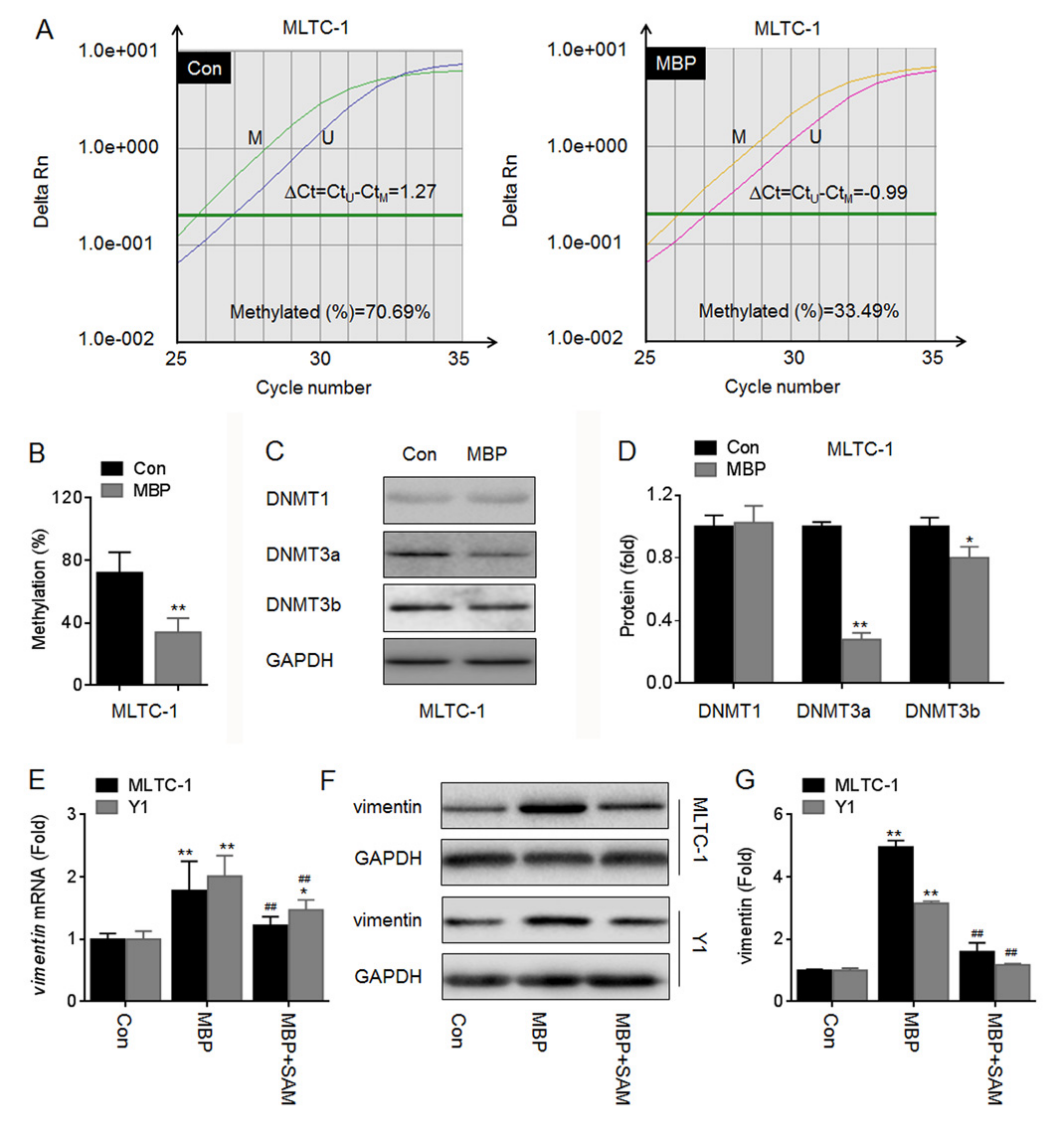
MBP improved the expression of vimentin by DNA demethylation. (A-D), MLTC-1 cells were exposed to 1000 nM MBP for 24 h, the methylation status of vimentin promoter was determined in triplicate by qMSP (A and B). Annotation, methylated (M); unmethylated (U); the percentage of methylation in a sample was estimated using the following formula: methylation (%) = (M/M+U) ×100% = [1/(1+U/M)] ×100% = [1/(1+2^∆Ct^)] ×100%. (C and D), Western blots analysis and relative protein levels of DNMT1, DNMT3a, and DNMT3b. *p<0.05 and **p<0.01 compared with medium control cells. (E-G), MLTC-1 and Y1 cells were exposed to 1000 nM MBP in the presence or absence of 200 μM SAM for 24 h, respectively. (E) qRT-PCR analysis in triplicate of *vimentin* mRNA. (F) Western blots analysis and (G)relative protein levels of vimentin. *p<0.05 and **p<0.01 compared with medium control cells; ^##^p<0.01 compared with cells exposed to MBP alone.

Analyzing by BLAST (http://blast.ncbi.nlm.nih.gov) and MethPrimer (http://www.urogene.org), we found that the sequences “CGGGCTTTCC” in the vimentin promoter is similar to kappaB DNA elements (GGGRNYYYCC), and that these sequences were located in the CpG islands (S2A Fig). Previous studies indicate that the DBP and/or MBP can cause the DNA demethylation [18, 19]. So we firstly investigated if the DNA demethylation was involved in the MBP-induced increased expression of vimentin. Our data showed that MBP decreased the average methylation level in vimentin promoter (Fig 6A and 6B). Changes in the methylation status are correlated with changes in DNA methyltransferase (DNMTs) activity [20]. Here, MBP decreased the expressions of DNMT3a and DNMT3b (two de novo methylation regulators [20]) in MLTC-1 cells (Fig 6C and 6D). Further, hypermethylation treatment by SAM dramatically blocked the MBP-induced elevation of vimentin mRNA and protein (Fig 6E–6G). These results suggested that the inhibition of de novo methylation might be involved in the DBP/MBP-induced elevation of vimentin.

**Fig 7:**
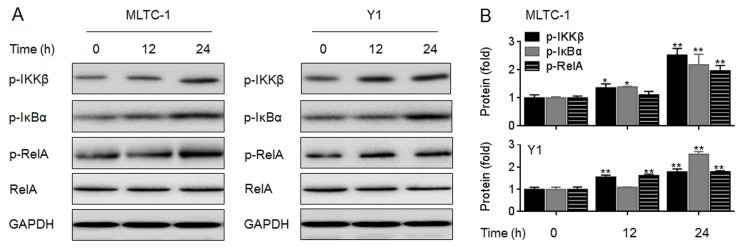
Effects of MBP on the activation of NF-κB. MLTC-1 and Y1 cells were exposed to 1000 nM MBP for 0, 12, or 24 h, respectively. (A) Western blots analysis and (B)relative protein levels of p-IKKβ, p-IκBα, and p-RelA. *p<0.05 and **p<0.01 compared with medium control cells.

**Fig 8:**
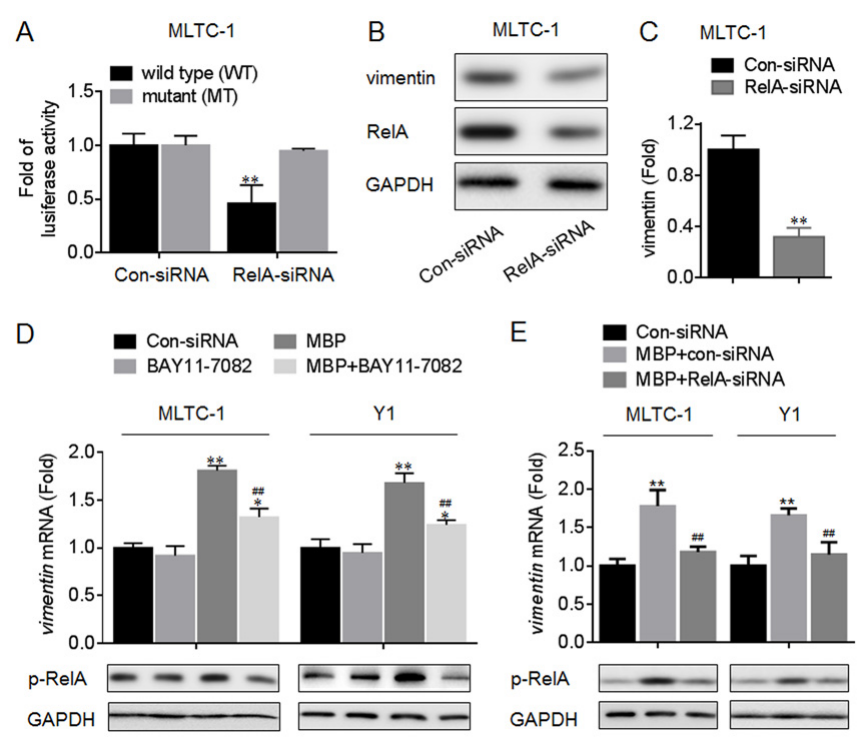
Functions of NF-κB in the transcriptional activation of vimentin. (A) MLTC-1 cells were co-transfected by Con-siRNA or RelA-siRNA plus pGL3-vimentin-Luc construct (wild type, WT; or mutant, MT) for 12 h. Luciferase reporter assay analysis of the effects of NF-κB on the transcriptional activity in *vimentin* promoter. (B and C) MLTC-1 cells were transfected by Con-siRNA or RelA-siRNA for 12 h. (B) Western blots analysis and (C)relative protein levels of vimentin. **p<0.01 compared with cells transfected by Con-siRNA. (D) After MLTC-1 and Y1 cells were pre-treated by 0 or 10 μM BAY11-7082 for 12 h, they were exposed to 0 or 1000 nM MBP for 24 h. (D, top) qRT-PCR analysis in triplicate of *vimentin* mRNA, *p<0.05 and **p<0.01 compared with medium control cells; ^##^p<0.01 compared with cells exposed to MBP alone. (D, bottom) Western blots analysis of the expression of p-RelA. (E) After MLTC-1 and Y1 cells were pre-transfected by Con-siRNA or RelA-siRNA for 12 h, they were exposed to 1000 nM MBP for 24 h. (E, top) qRT-PCR analysis in triplicate of *vimentin* mRNA, **p<0.01 compared with medium control cells; ^##^p<0.01 compared with cells exposed to MBP plus Con-siRNA.

We next detected if NF-κB is involved in the elevation of vimentin and progesterone secretion induced by MBP. Our data showed that in MBP-treated MLTC-1 and Y1 cells, there were increased phosphorylations of IKKβ, IκBα, and RelA (the classical NF-κB signaling, Fig 7A and 7B). Then we determined the functions of NF-κB in the transcriptional activation of vimentin. The pGL3-vimentin-Luc constructs (wild type, WT or mutated, MT) were exhibited in S2B Fig. Luciferase reporter assay showed that, co-transfected with pGL3-vimentin-Luc construct (WT, but not MT) plus RelA-siRNA led to a significant decrease of the luciferase activity (Fig 8A). Moreover, knockdown of RelA decreased the expression of vimentin (Fig 8B and 8C) but not StAR (S3 Fig). Based on these data, we hypothesized that the classical NF-κB signaling was involved in the MBP-induced elevation of vimentin. So we used BAY11-7082 (an IKKβ-IκBα-RelA inhibitor) and RelA-siRNA to confirm our hypothesis. Our data showed that, inhibition of NF-κB activity or knockdown of RelA attenuated the MBP-induced increased expression of *vimentin* mRNA (Fig 8D and 8E). Collectively, these results suggested that the DNA demethylation and NF-κB activation were involved in the MBP-induced transcriptional elevation of vimentin.

### MBP enhanced the progesterone secretion via NF-κB/vimentin

**Fig 9:**
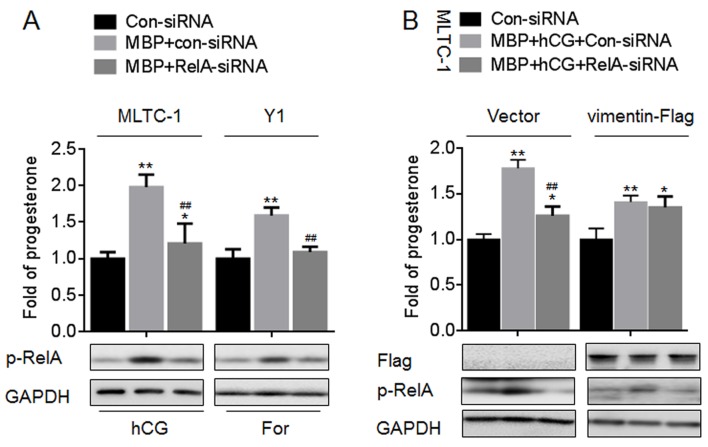
Effects of NF-κB/vimentin signaling on the MBP-induced progesterone secretion *in vitro*. (A) MLTC-1 and Y1 cells were treated as described in Fig 8E, the secretion of progesterone was determined in triplicate (top), and the expression of p-RelA was determined by Western blot (bottom). The amounts of progesterone measured in control cells were MLTC-1: 8.23±0.24 ng/ml, Y1: 9.42±0.37 ng/ml, respectively. (B) After MLTC-1 cells were co-transfected by Con-siRNA or RelA-siRNA plus Vector or vimentin-Flag construct for 12 h, they were exposed to 1000 nM MBP for 24 h. The secretion of progesterone was determined in triplicate (top), and the expressions of Flag and p-RelA was determined by Western blot (bottom). The amounts of progesterone measured in control cells were MLTC-1 (vector): 9.02±0.31 ng/ml and MLTC-1 (vimentin-FLag): 10.56±0.53 ng/ml, respectively. *p<0.05 and **p<0.01 compared with medium control cells; ^##^p<0.01 compared with cells exposed to MBP plus Con-siRNA.

Then we further determined the functions of NF-κB/vimentin signaling in the MBP-induced progesterone secretion. As NF-κB was an up-stream regulator of vimentin, knockdown of RelA decreased the secretion of progesterone in MLTC-1 and Y1 cells treated by hCG or For in the presence of MBP (Fig 9A). Next, we constructed the vimentin overexpression cells to further determine the functions of NF-κB/vimentin signal in the steroidogenesis induced by MBP. In vector-transfected MLTC-1 cells, knockdown of RelA attenuated the progesterone secretion induced by MBP plus hCG, however, in vimentin-Flag-transfected cells, this phenomenon was disappeared (Fig 9B). These results indicated that NF-κB/vimentin signaling was involved in the MBP-enhanced progesterone secretion.

### Effects of NF-κB/vimentin on DBP-induced testosterone secretion *in vivo*

**Fig 10:**
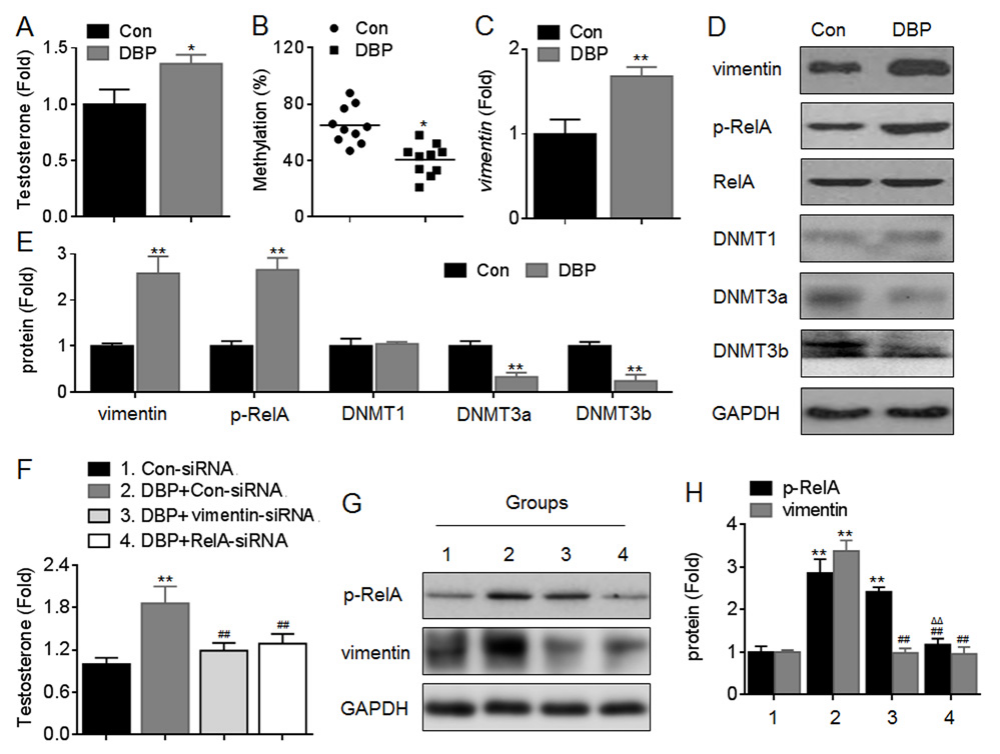
Effects of NF-κB/vimentin on DBP-induced testosterone secretion *in vivo*. The pubertal male, Sprague-Dawley rats (10 per guoup) were orally administered DBP at the doses of 1 mg/kg·day for 30 days. (A) The serum testosterone level was determined in triplicate. (B) The methylation status of vimentin promoter regions was determined in triplicate by qMSP. (C) qRT-PCR analysis in triplicate of the expression of *vimentin* mRNA. (D) Western blots analysis and (E) relative protein levels of p-RelA, vimentin, DNMT1, DNMT3a, and DNMT3b. *p<0.05 and **p<0.01 compared with rats exposed to no DBP. (F-H) After Con-siRNA, RelA-siRNA, or vimentin-siRNA was injected into the interstitial tissue of testis, respectively, the rats were orally administered DBP as described above. (F) The serum testosterone level was determined in triplicate. The amounts of testosterone measured in control animals were 16.36±0.22 ng/ml. (G) Western blots analysis and (H) relative protein levels of p-RelA and vimentin. **p<0.01 compared with Con-siRNA group; ^##^p<0.01 compared with DBP plus Con-siRNA group; ^ΔΔ^p<0.01 compared with DBP plus vimentin-siRNA group.

Finally, we used a DBP-treated *in vivo* model to confirm our conclusion. The pubertal male, Sprague-Dawley rats (10 per guoup) were orally administered DBP at the doses of 1 mg/kg·day for 30 days. At the end of then, the serum testosterone level was determined, the animals were sacrificed, and the Leydig cells were isolation and cultured as described previously [21]. Our data showed that, DBP increased the serum testosterone secretion significantly (Fig 10A). Further, DBP decreased the methylation level in *vimentin* promoter (Fig 10B), and inhibited the expressions of DNMT3a/b, but increased the expressions of vimentin and p-RelA in Leydig cells (Fig 10C–10E).

Then, we used siRNA against vimentin and siRNA against RelA to obtain the data to show the linkage of signaling of NF-κB-vimentin, and their contributions to the DBP-induced steroidogenesis. After the Con-siRNA, vimentin-siRNA, or RelA-siRNA was injected into the interstitial tissue of testis, respectively, the rats were orally administered DBP as described above. Our data showed that, knockdown of either vimentin or RelA attenuated the DBP-induced serum testosterone secretion (Fig 10F). Interestingly, knockdown of RelA decreased the DBP-induced increased expression of vimentin in Leydig cells, however, knockdown of vimentin had no significant effect on the RelA phosphorylation after DBP exposure (Fig 10G and 10H). Collectively, these data suggested that NF-κB/vimentin signaling was involved in the DBP-induced steroidogenesis *in vivo*.

## Discussions

Sexual maturation is the culmination of a complex sequence of events that leads to activation of the gonadotropic axis [22]. Abnormally precocious sexual development has been defined as the occurrence of Tanner stage B2 before 8 years in girls and Tanner stage G2 before 9 years in boys [23], which characterised by rapid growth and skeletal advancement leading to the paradox of a tall child becoming a short adult secondary to early epiphyseal-fusion [24]. In some cases, it may develop in a child with advanced somatic maturation, premature adrenarche, premature ihelarche and premature menarche [22, 24]. Children with precocious puberty tend to seek the company of children of the same height and strength and may experience stress because of dyssynchrony in age and body appearance, peer rejection and poor self-image [25].

In the last 1–2 decades, the ages at pubertal onset have been decreased in the USA and Europe [26]. Studies indicates that the changes in lifestyle and adverse environmental factors must be the primary reasons [26], in particular, the widespread presence of EDCs has caught our attention as contributing to the trend of earlier onset of puberty [27, 28]. Some studies reported that effects of EDCs can be biphasic with low doses causing early puberty and high doses delayed puberty such as shown using triphenyltin in the female rat and phthalates in the male [27, 28]. Here, our main findings demonstrated that MBP exhibited stimulating effect apparently on steroidogenesis at low doses. Furthermore, we examined the expressions of proteins involved in steroid biosynthesis pathway, such as StAR, P450scc, 3β-HSD and vimentin and showed that the levels of StAR and vimentin were elevated by MBP in accordance with the steroidogenesis, however, MBP did not have impact on P450scc, 3β-HSD, β-actin, and tubulin.

In the male endocrine system, steroidogenesis occurs mainly in testis Leydig cells and adrenal cortex cells. Here, the mouse Leydig tumor cells (MLTC-1) and Murine Y1 adrenocortical tumor cells (Y1) cells were chosen for *in vitro* study because these two cells were widely used to determine the steroidogenesis for their abilities to produce progesterone, an intermediate product of steroidogenesis [15, 16]. The steroid biosynthesis process consists of two steps: 1, cholesterol transportation from "cholesterol pool" in cytoplasm to outer membrane of mitochondria and 2, cross transportation from outer to inner membrane of mitochondria [15–17, 29]. StAR has been generally accepted as a key protein in the delivery of cholesterol cross the mitochondrial membrane [17]. In addition, vimentin, one of the cell skeleton proteins, was considered to be the main protein which involved in conveying cholesterol from "cholesterol pool" in cytoplasm to outer membrane of mitochondria [11, 30]. In steroidogenic cells, the cholesterol for steroidogenesis is stored in lipid droplets and vimentin intermediate filaments directly contact with mitochondria and lipid droplets [12, 31, 32]. So, vimentin is thought to be a bridge between cholesterol and mitochondria. In our present study, we found that, the hCG and/or forskolin significantly improved the expression of StAR but not vimentin. So we suggested that the progesterone production induced by hCG and/or forskolin was mediated by the increased expression of StAR. However, MBP significantly elevated the expressions of vimentin. So we suggested that the progesterone production induced by MBP was mediated by the increased expression of vimentin. So, the progesterone production is reduced with vimentin knockdown only with MBP stimulation and not with hCG and/or forskolin. Further, knockdown of vimentin blocked the MBP-induced progesterone biosynthesis, in which, a novel molecular mechanism that the DNA demethylation and NF-κB activation were involved.

DNA methylation is a process mediated by DNA methyltransferases via which methyl groups are covalently added to the 5’-position of cytosine in the CpG dinucleotide, causing the suppression of gene expression [33]. The modification of DNA by methylation alters gene transcription by either blocking the access of certain transcription factors to their consensus sequences on the promoter region, or by allowing the binding of methyl-CpG-binding proteins that recognize methylated DNA and recruit protein partners to suppress gene expression [34]. Here we found that the sequences “CGGGCTTTCC” in the *vimentin* promoter is similar to kappaB DNA elements (GGGRNYYYCC), and that these sequences were located in the CpG islands. In this regard, we firstly determined the effects of MBP on the methylation status in the *vimentin* promoter via qMSP. Further, we investigated the effects of MBP on the expressions of DNMT1, DNMT3a, and DNMT3b. Here, MBP decreased the average methylation level in vimentin promoter and the expressions of DNMT3a and DNMT3b in MLTC-1 cells, suggesting that the inhibition of de novo methylation in *vimentin* promoter was occurred after cells were exposed to MBP. In addition, this process demethylation might be involved in the facilitating of binding of NF-κB to the kappaB DNA element in the *vimentin* promoter. So we then exposed MLTC-1 and Y1 cells to MBP, and used BAY11-7082 and RelA-siRNA to block the activation/expression of NF-κB. Our data showed that inhibition of NF-κB attenuated the MBP-induced increased expression of vimentin. Collectively, these data suggested that MBP improved the expression of vimentin by inducing the DNA demethylation and NF-κB activation.

In summary, our *in vitro* and *in vivo* study demonstrated that MBP can alter steroid production by the mechanism that mainly involves in the elevation of vimentin. Indeed, MBP induced the DNA demethylation in the *vimentin* promoter, and induced the activation of NF-κB. These two processes enhanced the expression of vimentin synergistically, and mediated the MBP-induced steroid production.

## Supporting Information

S1 Fig Effects of MBP on the cell viability. The mouse (A) MLTC-1 and (B) Y1 cells were exposed to 0 ~ 106 nM MBP as indicated for 24 h, the cell viabilities were evaluated in triplicate by WST-8 hydrolysis using a Cell Counting Kit-8 assay. *p < 0.05 compared with medium control cells.
(TIF)

S2 Fig Description of the vimentin promoter. (A) schematic illustration that the sequences “CGGGCTTTCC” in the vimentin promoter is similar to kappaB DNA elements (GGGRNYYYCC), and that these sequences were located in the CpG islands. (B) The pGL3-vimentin-Luc constructs (wild type, WT or mutated, MT).
(TIF)

S3 Fig Effects of NF-κB on the expression of StAR. MLTC-1 cells were transfected by Con-siRNA or RelA-siRNA for 12 h. (A) Western blots analysis and (B)relative protein levels of StAR.
(TIF)
