# Supplementary figures and images for: Vimentin-Mediated Steroidogenesis Induced by Phthalate Esters: Involvement of DNA Demethylation and Nuclear Factor κB
Source: PLoS One. 2016 Jan 8;11(1):e0146138. doi: 10.1371/journal.pone.0146138 (PMC4706347; doi:10.1371/journal.pone.0146138)

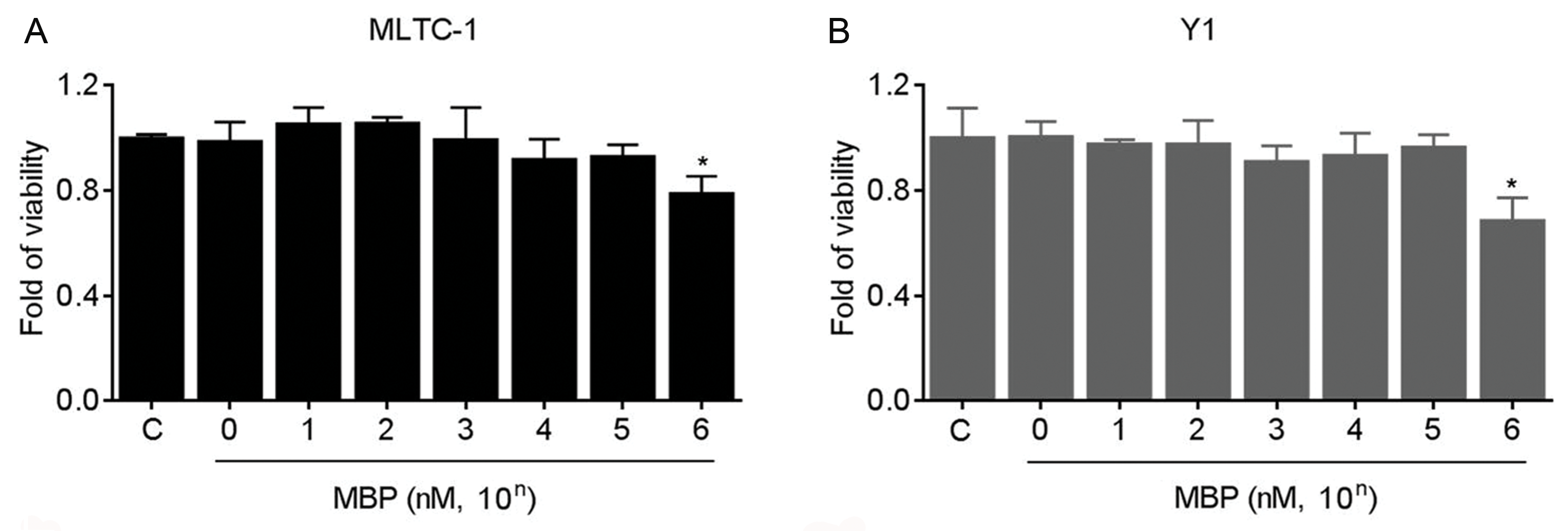

Supplement: S1 Fig — The mouse (A) MLTC-1 and (B) Y1 cells were exposed to 0 ~ 106 nM MBP as indicated for 24 h, the cell viabilities were evaluated in triplicate by WST-8 hydrolysis using a Cell Counting Kit-8 assay. *p < 0.05 compared with medium control cells. (TIF) [file pone.0146138.s001.tif]

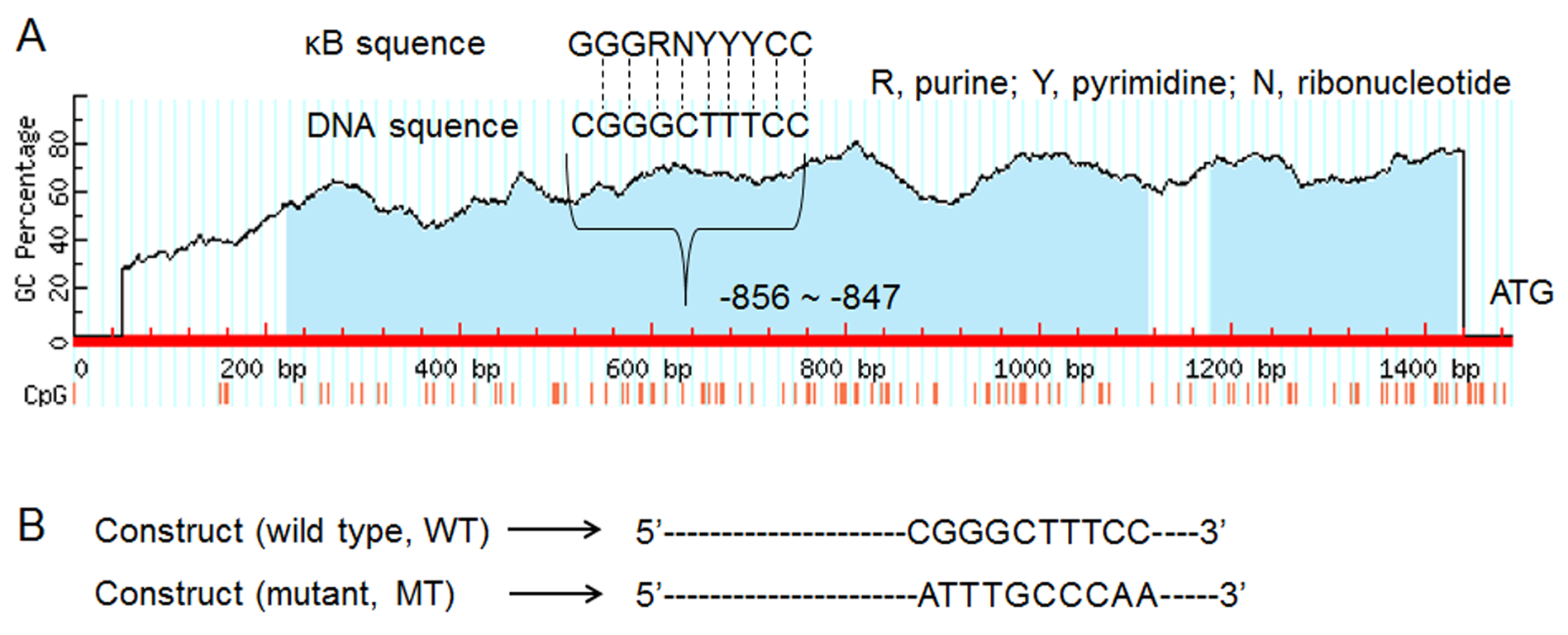

Supplement: S2 Fig — (A) schematic illustration that the sequences “CGGGCTTTCC” in the vimentin promoter is similar to kappaB DNA elements (GGGRNYYYCC), and that these sequences were located in the CpG islands. (B) The pGL3-vimentin-Luc constructs (wild type, WT or mutated, MT). (TIF) [file pone.0146138.s002.tif]

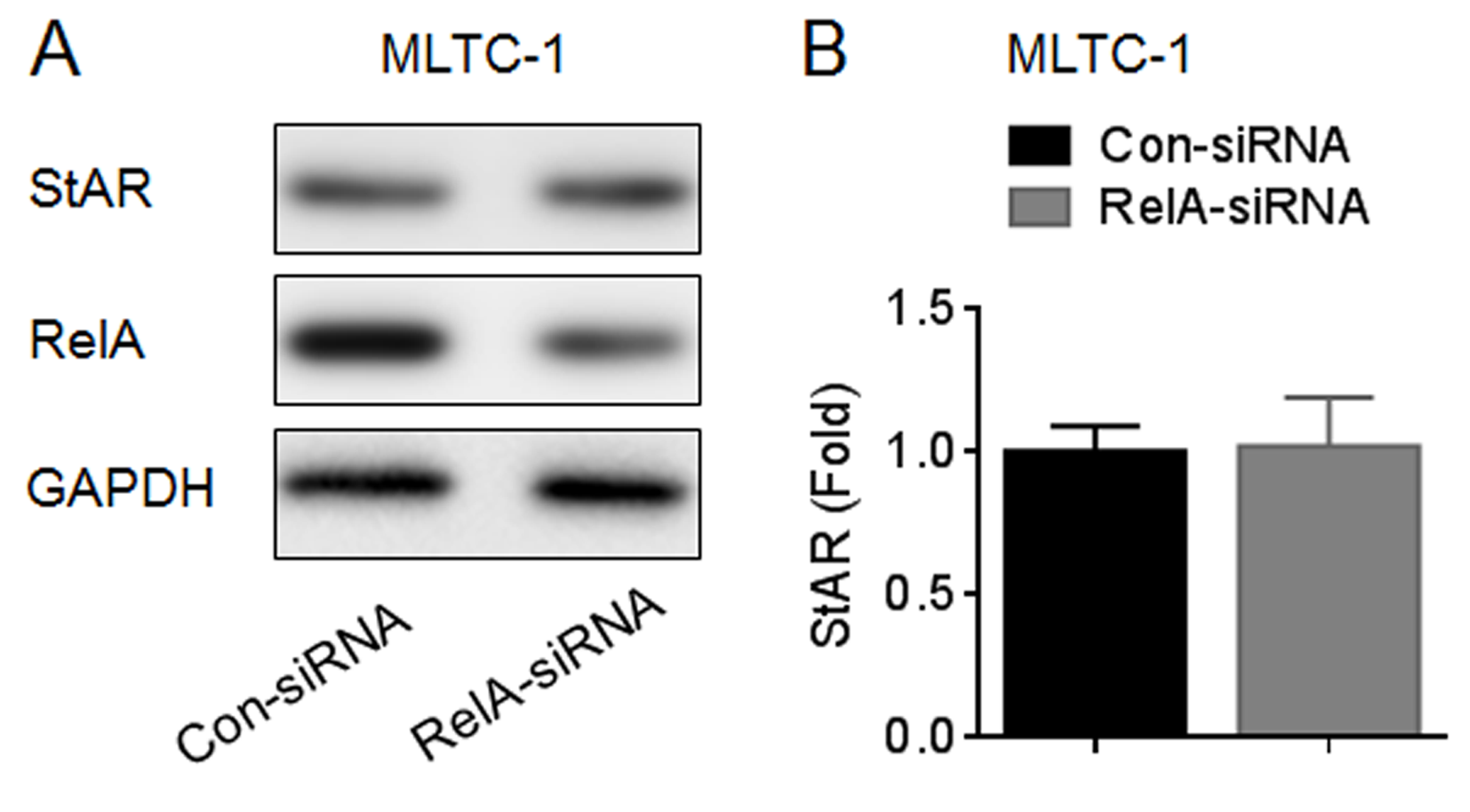

Supplement: S3 Fig — MLTC-1 cells were transfected by Con-siRNA or RelA-siRNA for 12 h. (A) Western blots analysis and (B)relative protein levels of StAR. (TIF) [file pone.0146138.s003.tif]
